# Pyometra Perforation Caused by *Actinomyces* without Intrauterine Device Involvement

**DOI:** 10.1155/2013/658902

**Published:** 2013-05-16

**Authors:** Hideharu Hagiya

**Affiliations:** Emergency Unit and Critical Care Center, Tsuyama Central Hospital, 1756 Kawasaki, Tsuyama, Okayama 708-0841, Japan

## Abstract

An 86-year-old woman with diabetes mellitus and severe decubitus at the sacral and calcaneal regions stemming from poor daily activity was diagnosed with pyometra perforation caused by *Actinomyces*. No foreign materials, including an IUD, were found inside the uterus. Pyometra is usually caused by Enterobacteriaceae or anaerobes derived from the gastrointestinal tract. The virulence of *Actinomyces* is rather low, and, in almost all the reported cases of *Actinomyces*-related pyometra, an intrauterine device (IUD) was involved. Although rare, *Actinomyces* may be ascribed as a virulent pathogen that causes pyometra in the absence of foreign materials.

## 1. Introduction

Pyometra is caused by bacterial infection of the uterus, which frequently occurs in postmenopausal women. It can vex physicians by causing fever of unknown origin, or sometimes, panperitonitis due to perforation. Pathogens considered common are Enterobacteriaceae or anaerobes derived from the gastrointestinal tract. *Actinomyces* has been reported as the causative pathogen; however, in almost all reported cases, an intrauterine device (IUD) was involved [1–4]. I describe a case of panperitonitis caused by *Actinomyces*-induced pyometra perforation without IUD involvement.

## 2. Case

**Figure 1:**
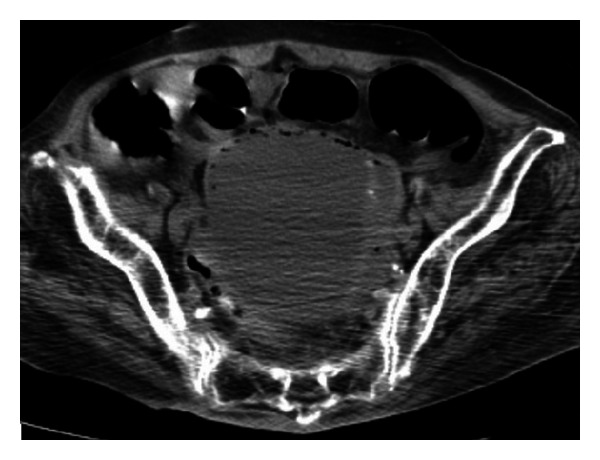
Emphysematous changes inside the wall of the distended uterus.

 An 86-year-old woman with diabetes mellitus and severe decubitus at the sacral and calcaneal regions stemming from poor daily activity showed altered mental status. She has never used IUD previously. On arrival at our hospital, she was in shock, and physical examination revealed tenderness over the lower abdomen. A laboratory examination revealed pleocytosis (white blood cell count, 24,400/*μ*L) and a high inflammatory activity (C-reactive protein level, 8.5 mg/dL). Her serum glucose level was 425 mg/dL and hemoglobin A_1_c level was 8.2%. Ultrasonography showed fluid retention at Douglas' pouch, and abdominal computed tomography revealed gas production inside her distended uterus and intra-abdominal fluid retention (Figure 1). Under a diagnosis of pyometra perforation, an emergent total abdominal hysterectomy and bilateral salpingo-oophorectomy was performed. Purulent ascites accounted for intraperitoneal space and the perforation site was found at the fundus of the edematous, swollen, and fragile uterine. Microscopic examination showed an infiltration of many neutrophils with necrotic changes; phlegmonous or gangrenous inflammatory changes, but no malignant findings were obtained. No IUD was detected inside the uterus. Gram staining of intraperitoneal and uterine pus revealed a polymicrobial pattern containing a filamentous organism, but bacterial culture detected only *Actinomyces*. The patient was successfully treated with meropenem for 8 days, followed by cefmetazole for 7 days, and was discharged on hospital day 27. A detailed identification of the *Actinomyces* was not performed.

## 3. Discussion

*Actinomyces *is a nonmotile, nonspore-forming, nonacid-fast, Gram-positive, pleomorphic, anaerobic-to-microaerophilic filamentous bacillus [5]. It is prominent among the normal flora of the oral cavity but is less common in the lower gastrointestinal tract and female genital tract. Because the growth rate and virulence of *Actinomyces* are low, this organism requires the presence of broken or damaged mucous membranes or tissues to invade deeper structures and cause disease in humans.

Although many cases of abscess formation caused by *Actinomyces* at other sites of the body have been reported, only a few cases of *Actinomyces*-induced pyometra in humans that did not involve foreign materials or IUDs have been reported [6, 7]. The presence of foreign materials such as an IUD inside the uterus could promote the proliferation of *Actinomyces* through damage of the endometrial tissue. Malignant cells invade the endometrial tissue and can be a predisposing factor of *Actinomyces* infection; however, pathological examination did not reveal such a condition in the present case. 

*Actinomyces* usually causes a polymicrobial infection, with isolates containing as many as 5–10 bacterial species [8]. In this case, only *Actinomyces* was isolated, but Gram staining revealed other Gram-positive or Gram-negative organisms. Coinfection with other obligate anaerobes was considered to have occurred, and the presence of these companion bacteria could have enhanced the relatively low virulence of *Actinomyces*, causing pyometra and the eventual perforation without IUD involvement. 

Although rare, *Actinomyces* may be ascribed as a virulent pathogen that causes pyometra in the absence of foreign materials.
